# Getting more bang for their buck: BCL2 inhibitors boost dendritic-cell function to enhance anti-cancer immune surveillance

**DOI:** 10.1186/s12967-024-04961-x

**Published:** 2024-03-28

**Authors:** Alfredo E. Montes-Gómez, Stephen W. G. Tait

**Affiliations:** 1 Cancer Research UK Scotland Institute, Switchback Road, Glasgow, G61 1BD UK; 2 School of Cancer Sciences, University of Glasgow, Switchback Road, Glasgow, G61 1BD UK

**Keywords:** Dendritic cells, BCL-2, Venetoclax, BH3 mimetics, Immune checkpoint inhibitors, DC maturation, Interferons

## Abstract

The anti-apoptotic BCL-2 protein family regulates cancer cell survival, thus it represents an important therapeutic target. Indeed, a drug class, called BH3-mimetics, have been developed to directly target BCL2 proteins and promote cancer cell death. Conventional wisdom suggests that the primary anti-cancer effect of BCL-2 inhibition is through induction of cancer cell death. However, a recent study by Zhao and colleagues describes that BCL-2 inhibition also enhances the function of classical dendritic cells, unleashing their role in immunosurveillance, promoting T cell immunity and tumour regression. Thus, inhibiting anti-apoptotic BCL-2 function may have a multi-pronged anti-tumour action.

One of the most recent successes in clinical oncology are immune checkpoint inhibitors (ICI), which harness anti-tumour T-cell function, however, clinical translation of cancer immunology seldom focuses on antigen presenting cells. In the current study, Zhao and colleagues investigated ways to enhance the activity of antigen presenting dendritic cells (DC) that are the main orchestrators of T cell function [1].

A robust and effective T response relies on efficient antigen presentation by DCs that licenses T cells to eliminate tumour or infected cells [2]. Utilising conditionally immortalised immature dendritic cells (de-iniDCs), the authors applied a genome-wide CRISPR screen to identify genes that improved DC-mediated antigen-presentation [1]. Intriguingly, this screen identified various apoptotic genes as putative immune checkpoint regulators in DC function. The authors focused on anti-apoptotic BCL-2 for which a clinically approved BH3-mimetic, called venetoclax, is available [3].

Intriguingly, inhibiting BCL-2 function -either through genetic deletion or venetoclax treatment- promoted DC function as determined by several criteria, including improved maturation and antigen presentation to T cells. Upon BCL-2 inhibition, a strong type 1 interferon (IFN-I) response was observed, which is known to promote DC function [4]. Towards in vivo application of these findings, the authors found that venetoclax treatment sensitised lung tumours to immune-checkpoint blockade (anti-PDL1) thereby promoting tumour regression. Investigating the anti-tumour immune response, they observed, strong evidence of intra-tumoral cDC1 cell maturation and T cell activation. Adoptive transfer of de-iniDCs pre-treated with venetoclax or devoid of BCL2 and stimulated with tumour lysates also showed enhanced maturation and tumour infiltration with almost complete tumour regression in the presence of anti-PD1. To corroborate a role for cDC1s in tumour immunosurveillance, tumour-bearing lethally irradiated mice were reconstituted with wild-type bone marrow or bone marrow lacking type 1 cCDs (from *Batf3*^*−/−*^ KO donor mice). BCL2-inhibition controlled tumour growth in the animals reconstituted with WT cells but failed to do so in the case of the cDC1 depleted bone marrow. Towards clinical translation of these findings, cDC1s isolated from acute myeloid leukaemia (AML) patients, treated with a combination of venetoclax and azacytidine, displayed higher levels of DC migration and maturation markers. The authors do not show the effect of venetoclax on the tumours cells, hence we cannot rule out that tumour cells may die via immunogenic cell death or secrete antigens that also could enhance DC function.

Collectively, these data show that inhibition of BCL2 enhances the function of cDC1s to mediate anticancer effects, unveiling a synergistic effect with PD-1 blockade. Therefore, antagonism of BCL-2 function can have multiple anti-tumourigenic effects, extending beyond direct killing of tumour cells. A central, outstanding question remains—*how does inhibition of BCL-2 function mediate these effects?* Given that cDCs remain viable, the activating effects of BCL-2 inhibition must be independent of its canonical role in regulation of cell viability. In the current study the authors find increased levels of cytosolic mtDNA upon BCL-2 inhibition. We and others have previously reported mtDNA release following mitochondrial permeabilization through BCL2 inhibition with BH3 mimetics [5, 6],congruent with this, it has been recently reported that besides BCL2 inhibition, TFAM loss (a histone-like protein that maintains mtDNA structure) also leads to mtDNA release, enhancing DC activation and the anti-tumour T cell response [7]. Kollhapp et al. reported that venetoclax plus anti-PD1 treatment promote anti-tumour immunity in a subcutaneous cancer model and observe that the CD8+T cell effector memory compartment is enriched by venetoclax as this population increases the expression of BCL-xl [8]

Regardless of underlying mechanism, this study provides compelling evidence that BCL-2 inhibition has diverse anti-tumourigenic effects beyond its modus operandum of tumour cell toxicity (Fig. 1), offering new possibilities to maximise the therapeutic potential of targeting BCL-2 function.

**Fig. 1:**
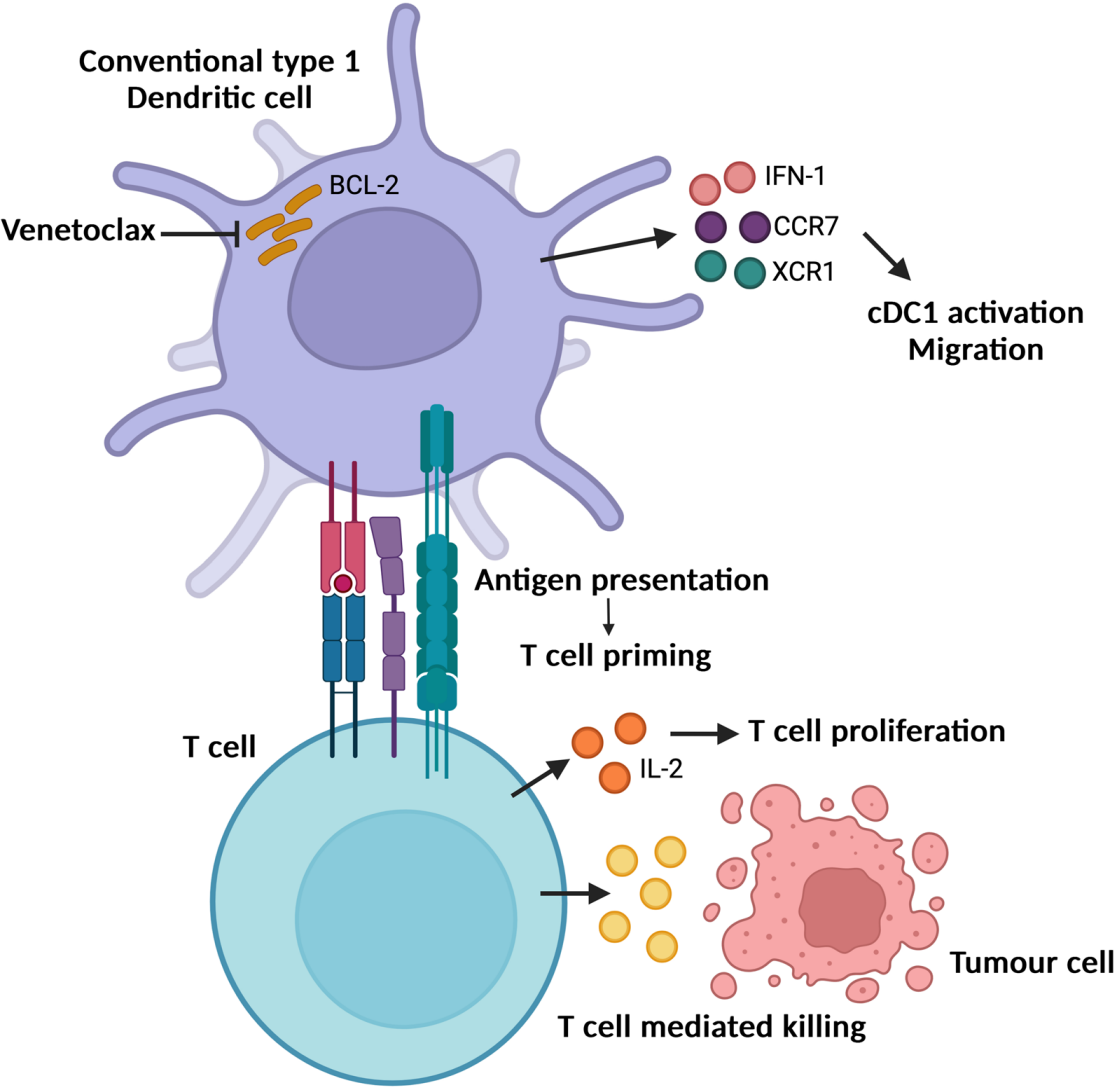
BCL-2 inhibition promotes anti-tumorigenic dendritic cell function (see text for details)

## Data Availability

Not applicable.

## Abbreviations

BCL-2: B cell lymphoma
ICI: Immune checkpoint inhibitors
DC: Dendritic cell
BMDC: Bone Marrow derived Dendritic Cell
MHC: Major Histocompatibility Complex
TCR: T cell receptor
De-iniDC: Immortalized immature Dendritic Cell
cDC: Classical Dendritic Cell
AML: Acute myeloid leukaemia
mtDNA: Mitochondrial DNA
MOMP: Mitochondrial Outer Membrane Permeabilization
TFAM: Mitochondrial Transcription Factor A
